# Genomic insights into antimicrobial potential and optimization of fermentation conditions of pig-derived *Bacillus subtilis* BS21

**DOI:** 10.3389/fmicb.2023.1239837

**Published:** 2023-09-29

**Authors:** Di Wu, Linglong Fu, Yunhe Cao, Na Dong, Defa Li

**Affiliations:** State Key Laboratory of Animal Nutrition, College of Animal Science and Technology, China Agricultural University, Beijing, China

**Keywords:** *Bacillus subtilis*, antimicrobial activity, secondary metabolites, fermentation conditions, response surface methodology, antibiotic alternative

## Abstract

*Bacillus* spp. have been widely used as probiotic supplements in animal feed as alternatives to antibiotics. In the present study, we screened a *Bacillus subtilis* strain named BS21 from pig feces. Antimicrobial activities, whole genome mining and UHPLC-MS/MS analysis were used to explore its antimicrobial mechanism. Strain BS21 showed Significant growth inhibition against a variety of animal pathogens, including *Escherichia coli*, *Salmonella enterica Pullorum*, *Salmonella enterica Typhimurium*, *Citrobacter rodentium*, *Shigella flexneri* and *Staphylococcus aureus*. Seven gene clusters involved in antimicrobial biosynthesis of secondary metabolites were encoded by strain BS21 genome, including four non-ribosomal peptides (bacillibactin, fengycin, surfactin and zwittermicin A), one ribosomal peptide (subtilosin A), one dipeptide (bacilysin) and one polyketide (bacillaene). Among them, production of surfactin, fengycin, bacillibactin, bacilysin and bacillaene was detected in the supernatant of *B. subtilis* strain BS21. To develop the potential application of BS21 in animal production, medium components and fermentation parameters optimization was carried out using response surface methodology (RSM). Production of antimicrobial secondary metabolites of strain BS21 was increased by 43.4%, and the best medium formula after optimization was corn flour 2%, soybean meal 1.7% and NaCl 0.5% with optimum culture parameters of initial pH 7.0, temperature 30°C, rotating speed at 220 rpm for 26 h. Our results suggested that strain BS21 has the potential for large-scale production and application as a potential source of probiotics and alternative to antibiotics for animal production.

## 1. Introduction

Antibiotics have been added to animal feed to improve livestock production performance and reduce disease since the 1950s (Low et al., 2021). Extensive use of antibiotics leads to the development of resistant bacteria and residues in animal-derived food (Van et al., 2020). Safe and effective antibiotic alternatives are therefore urgently needed. Probiotics, one of the most promising antibiotic alternatives, are reported to act against pathogenic bacteria, improve immune system functions and restore the intestinal microbial balance of farm animals (Plaza-Diaz et al., 2019).

*Bacillus spp*. are important sources of probiotics in animal feed. The ideal *Bacillus* species applied in animals feed are usually isolated from gastrointestinal tracts and feces of different animals, such as pigs, chickens, ruminants and aquatic animals (Bahaddad et al., 2023). *B. subtilis*, *B. licheniformis*, *B. velezensis*, *B. coagulans*, and *B. amyloliquefaciens* have been used to feed animals and shown to exert beneficial effects on the host (Du et al., 2019; Li et al., 2019; Shang et al., 2022). Antimicrobial properties of probiotic strains are important to prevent infection caused by pathogenic bacteria, which is a functional property used to select potential probiotics (Fijan, 2023). In recent years, *Bacillus* species have been demonstrated to exhibit broad-spectrum activity against microbes, because they produce multiple antimicrobial metabolites, mainly antimicrobial peptides (AMPs) and polyketides (Chen et al., 2019).

AMPs from *Bacillus* can be divided into non-ribosomally synthesized peptides (NRPSs) or ribosomally synthesized peptides based on their synthesis pathway (Puan et al., 2023). Fengycins, iturins, surfactin, bacillibactin and bacitracin derived from *Bacillus* are all members of a class of molecules known as NRPSs. These NRPSs are commonly made by large, multisubunit enzymes, often known as non-ribosomal peptide synthetases (Shahid et al., 2021). These peptides are often cyclized, chained, or branched and can be further modified by N-methylation, glycosylation, acylation or heterocyclic ring formation (Riccardo et al., 2022). AMPs synthesized by ribosomes, including subtilin, bacthuricin and cerecin, commonly known as bacteriocins, exist as either post-translationally modified or unmodified peptides. These bacteriocins exhibit a range of activities, including cell lysis, quorum sensing mediation and induction of genetic competence (Rebuffat, 2020). In general, these AMPs are cationic and hydrophobic or amphiphilic, and the cellular membrane of bacteria, in most cases, is the main target for AMPs to exert antimicrobial activity (Zhang et al., 2021). In addition to anti-bacterial and anti-fungal properties, *Bacillus*-derived AMPs have also shown antiviral, antitumor and immunoregulatory activities, making them another attractive alternative to antibiotics in recent years (Caulier et al., 2019; Basi-Chipalu et al., 2022). Polyketides are another important antimicrobial compound produced by the *B. subtilis* group. *Bacillus* species secrete three antimicrobial polyketides and their variants (bacillaene, difficidin and macrolactin) exhibit antibacterial activities by selectively inhibiting protein synthesis. Moreover, these antimicrobial metabolites could have synergistic effects against pathogens (Chen et al., 2019).

The production of secondary metabolic products in microorganisms is influenced by the strain’s genetic traits as well as the nutritional and growth conditions (Puan et al., 2023). Response surface methodology (RSM) is a computational method used for the identification of interactions between response values and defined factors as well as the combinations of factors responsible for an optimal response (Zhou et al., 2023). RSM is commonly employed in food, chemistry and environmental engineering for improved efficiency of production and lower production costs (Yolmeh and Jafari, 2017).

In this research, we isolated a *B. subtilis* strain from pig feces named BS21 with broad-spectrum antibacterial activity. The genome of BS21 was sequenced and analyzed and a total of seven gene clusters of antimicrobial secondary metabolites were predicted, including six antimicrobial peptides (bacillibactin, fengycin, surfactin, zwittermicin A, subtilosin A and bacilysin) and one polyketide (bacillaene). In addition, we successfully detected surfactin, fengycin, bacillibactin, bacilysin and bacillaene from fermentation supernatant. Optimization studies using the RSM showed that optimal factors affected the maximal production of antimicrobial secondary metabolites from strain BS21, providing a theoretical basis for large-scale fermentation. This study will lay the foundation for researching molecular mechanisms of antimicrobial activity, and provide theoretical support that *B. subtilis* BS21 has the potential to be developed as an antibiotic alternative in animal production.

## 2. Materials and methods

### 2.1. Isolation and screening of strain BS21

The feces sample was collected from free-range pigs for isolation and screening of bacteria. Briefly, 1 g feces sample was homogenized in 10 mL normal saline (0.9% NaCl) by vortex mixture, 100 μL aliquot from selected tenfold serial dilutions were spreadly plated on Luria-Bertani (LB, Qingdao Hope Bio-technology Co., Ltd., Qingdao, China) solid medium (1% tryptone, 0.5% yeast extract, 1% NaCl and 1.5% agar). These plates were then incubated at 37°C for 18–24 h. Colonies with distinct morphology were selected and cultured in LB at 37°C for 24 h. Oxford cup method was used to test antimicrobial activity against *E. coli* of these bacteria. A potent isolate (BS21) was selected for further studies.

### 2.2. Microbial strains and growth conditions

Strain BS21 was stored at the China General Microbiological Culture Collection Center (No. CGMCC 20391). Pathogenic bacteria *Escherichia coli* (*E. coli*) K88, *E. coli* O127:H6, *Salmonella pullorum* (*S. pullorum*) CVCC1791, *Salmonella typhimurium* (*S. typhimurium*) SL1344, *Citrobacter rodentium* (*C. rodentium*) DBS100, *Shigella flexneri* (*S. flexneri*) 2457T, *Staphylococcus aureus* (*S. aureus*) CVCC1882 and CVCC43300 were acquired from the China Veterinary Culture Collection Center. All microbial strains were grown aerobically in LB medium with constant shaking (220 rpm) at 37°C.

### 2.3. Antimicrobial activity assay

*Bacillus subtilis* BS21 was cultured in LB medium with constant shaking (220 rpm) at 37°C for 24 h. Suspensions were centrifuged at 12,000 *g* for 15 min to remove bacteria, and the supernatant was filter sterilized using a 0.22 mm filter (Merck Millipore, Darmstadt, Germany) to obtain cell-free supernatant. Oxford cup method was used to test the antimicrobial activity of BS21 supernatant. Briefly, 100 mL of LB media agar was mixed with 10^8^ pathogenic bacteria to attain 10^6^ CFU/mL, and then the mixture was placed into petri dishes with Oxford cups (Mostafa et al., 2018; Xiang et al., 2021). Oxford cups were removed when LB media agar solidified. A volume of 150 μL of cell-free supernatant was dispensed into each well, followed by co-culturing alongside various pathogens at 37°C for 12 h, and then the diameters of the inhibition zone were measured.

### 2.4. Identification of strain BS21

The total DNA from strain BS21 was extracted through the use of a bacterial genomic DNA kit (CWBIO, Beijing, China) following the directions provided by the manufacturer. PCR amplification of the extracted DNA was conducted using the forward primer (5′-AGAGTTTGATCCTGGCTCAG-3′) and reverse primer (5′-GGTTACCTTG TTACGACTT-3′). Sequencing of the resultant PCR products was performed by Beijing Ruibio Biotech Co., Ltd. The obtained sequences were subjected to a BLAST search, and the selected homologous *Bacillus* sequences were aligned using MEGA 7.0 software. Subsequently, a phylogenetic tree was constructed based on neighbor-joining method.

### 2.5. Genomic DNA extraction

BS21 was grown in LB medium at 30°C for around 10 h, with a shaking speed of 220 rpm. Following centrifugation at 12,000 *g* for 15 min, the cells were collected. The total DNA was isolated from strain BS21 using a bacterial genomic DNA kit (CWBIO, Beijing, China). The isolated genomic DNA was quantified using a TBS-380 fluorometer (Turner BioSystems Inc., CA, USA).

### 2.6. Genome sequencing and assembly

Genomic DNA of strain BS21 was sequenced (Shanghai Majorbio Bio-pharm Technology Co., Ltd., Shanghai, China) using a combination of PacBio Sequel II and Illumina sequencing platforms. In preparation for PacBio sequencing, the DNA fragments were subjected to purification, end-repair and ligation with SMRT bell sequencing adapters. The sequencing libraries underwent triplicate purification. An approximately 10 kb insert library was constructed and sequenced using one SMRT Cell and standard methods. Additionally, DNA samples were fragmented into 400–500 bp fragments for Illumina sequencing. Libraries constructed via these sheared fragments were used for Illumina sequencing. The prepared libraries were then used for paired-end Illumina sequencing on an Illumina Novaseq6000 machine. The Illumina data were used to evaluate the complexity of the genome. The data generated from PacBio and Illumina platforms were used for bioinformatics analysis.

### 2.7. Genome annotation

The coding sequences (CDSs) of the bacterial genome were predicted using Prodigal v2.6.3^1^ and GeneMarkS.^2^ tRNA and rRNA genes were predicted by tRNA-scan-SE v2.0^3^ and Barrnap v0.9.^4^ In addition, genomic islands, prophages and CRISPR-Cas were predicted using Island-Viewer,^5^ PHAST^6^ and CRISPRFinder,^7^ respectively. The functions of genes were annotated using the COG (Clusters of Orthologous Genes) database through sequence alignment tools such as Diamond,^8^ BLAST+^9^ and HMMER.^10^ AntiSMASH v4.0.2^11^ was used to predict the secondary metabolite gene clusters in the genome of strain BS21.

### 2.8. UHPLC–MS/MS analysis of antimicrobial secondary metabolites

The crude extraction method of antimicrobial secondary metabolites refers to Al-Dhafri and Ching (2022) and Chen et al. (2018). Ammonium sulfate (Macklin Biochemical Technology Co., Ltd., Shanghai, China) was carefully added into the cell-free supernatant of *B. subtilis* BS21 to a final concentration of 70%. This was then allowed to stir overnight at 4°C. The precipitate was obtained by centrifugation at 12,000 *g* for 15 min followed by an ethanol extraction. Ethanol extract was subjected to rotating-drying at 45°C. Crude extract was dissolved in deionized water for further analysis.

An ultrahigh-performance liquid chromatography machine coupled to 6460 triple quadrupole mass spectrometry (UHPLC–MS/MS) (Agilent Technologies Inc., CA, USA) were used to detect secondary metabolites produced by strain BS21. UHPLC was conducted with a C18 column (100 × 2.1 mm, 1.7 μm) (Waters Corp., MA, USA). Eluent A and B were composed of H_2_O/0.1% formic acid (Macklin Biochemical Technology Co., Ltd., Shanghai, China) and CH_3_CN (Thermo Fisher Scientific Inc., Waltham, USA)/0.1% formic acid, separately. The flow rate was set at 300 μL/min, and the elution gradient ranged from 95% A/5% B at 0 min to 5% A/95% B at 60 min. For the 6460 triple quadrupole MS, the following conditions were applied: QQQ gas temperature of 350°C, gas flow rate of 10 L/min, sheath gas temperature of 350°C, sheath gas flow rate of 11 L/min and capillary voltage of 3500 V. Ions were accumulated using the MS2 scan mode with a scan time of 500 ms and a mass range between 200 and 2000 m/z.

### 2.9. Single-factor experiment

*Bacillus subtilis* BS21 was initially grown in 50 mL LB medium with constant shaking (220 rpm) at 30°C for 24 h. Single-factor experiment was used to select medium components (including different carbon sources, nitrogen sources, inorganic ions and their proportions) and fermentation parameters (temperature, initial pH, rotational speed and fermentation time). After each single factor was selected, the fermentation medium or parameter was formulated according to experimental needs. The diameter of inhibition zone was tested using Oxford cup method and the indicator pathogen was *E. coli* K88. The inhibition zone diameter was used to characterize the production of antimicrobial secondary metabolites.

### 2.10. Response surface methodology

#### 2.10.1. Plackett-Burman design (PBD)

Based on the data from single-factor experiment, the PBD was applied to analyze the main effect factors of medium composition: carbon source (corn flour), nitrogen source (soybean meal), mineral salts (NaCl) and fermentation parameters (fermentation temperature, initial pH, rotating speed and fermentation time). To perform the PBD, Design-Expert 13.0 software was used.

#### 2.10.2. Box–Behnken design (BBD)

To further improve the production of antimicrobial secondary metabolites, BBD of response surface methodology was used to evaluate the most optimum level, impact and interactions of the screened principal component factors (soybean meal, temperature and time). BBD was conducted using Design-Expert 13.0 software.

### 2.11. Growth curve of BS21 under optimum conditions

Strain BS21 was cultured in optimum medium (2% corn flour, 1.7% soybean meal and 0.5% NaCl) under optimum fermentation parameter (30°C, pH 7.0, 220 rpm). Samples were carried out every 4 h for 36 h and were diluted appropriately to count bacteria cell numbers in LB solid medium plates.

### 2.12. Statistical analyses

The data were analyzed by one-way analysis of variance using SPSS 23.0 statistical software (IBM Corp., Armonk, NY, USA). A Duncan’s test was employed in our testing for significant differences between treatments. Statistical differences of the mean value were defined at *p* < 0.05.

## 3. Results

### 3.1. Antimicrobial activity of strain BS21

**Figure 1:**
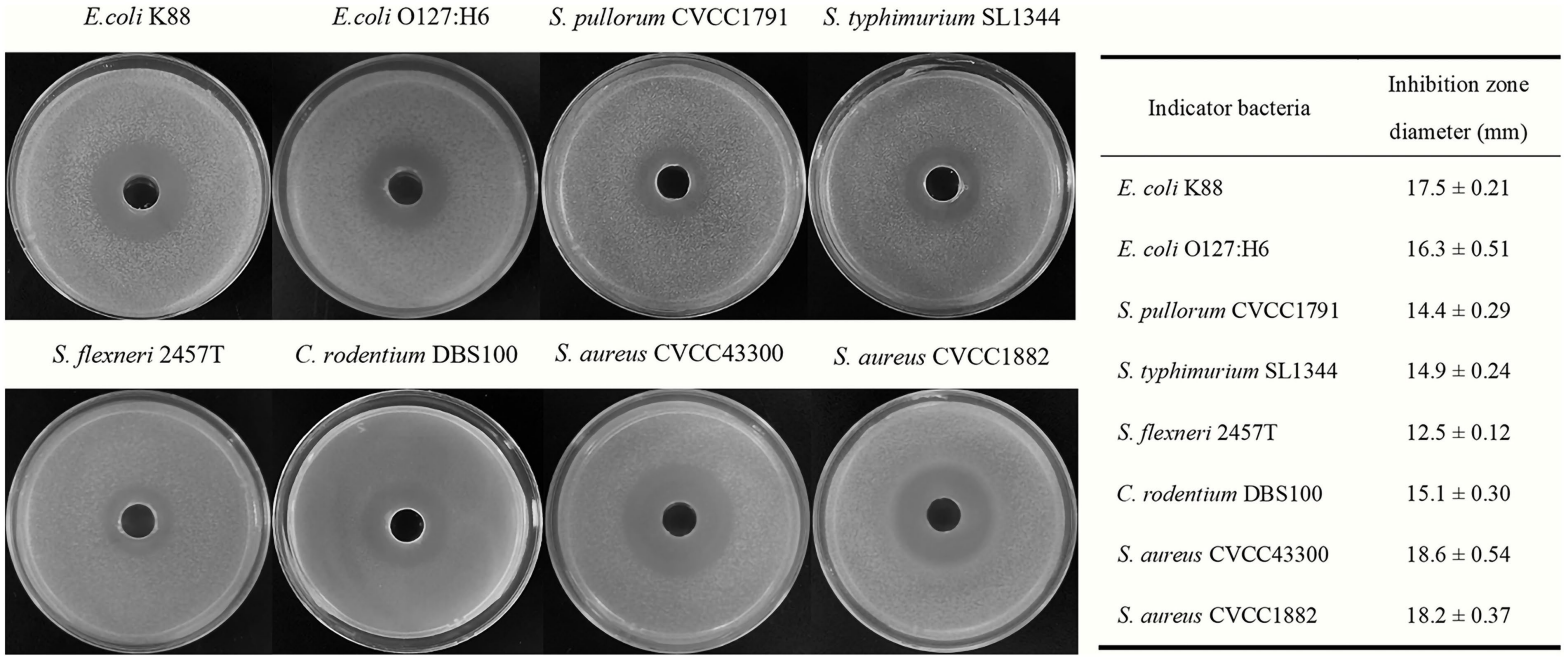
The antimicrobial activities of strain BS21 culture supernatant against different pathogens.

The antimicrobial activities of *Bacillus subtilis* BS21 against pathogens are shown in Figure 1. BS21 exhibited strong antimicrobial activities against Gram-negative pathogens, including *E. coli* K88, *E. coli* O127:H6, *S. pullorum* CVCC1791, *S. typhimurium* SL1344, *C. rodentium* DBS100 and *S. flexneri* 2457T. In addition, strain BS21 had an obvious inhibiting effect on Gram-positive pathogens (*S. aureus* CVCC1882 and *S. aureus* CVCC43300).

### 3.2. Strain identification

**Figure 2:**
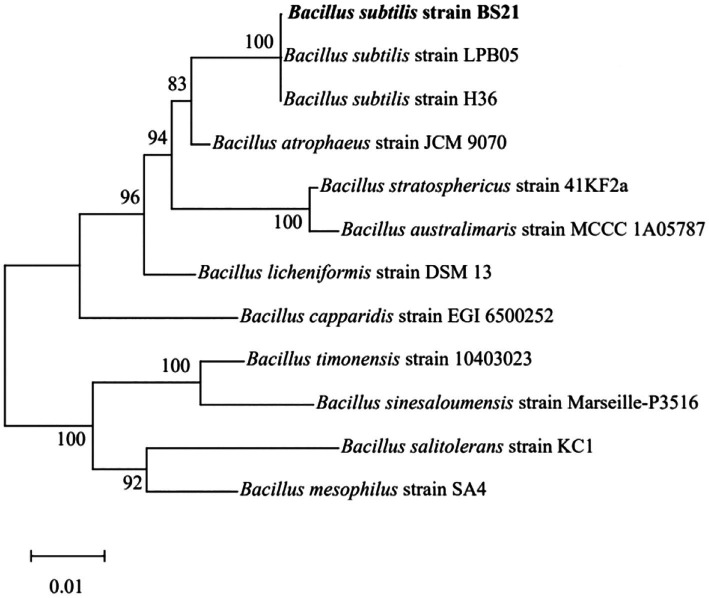
The neighbor-joining tree of strain BS21 based on 16S rRNA sequences.

The 16S rRNA sequence of strain BS21 showed 100% homology to *B. subtilis* strain NGS-STR-5 (GenBank number MF083067) and *B. subtilis* strain NG4-17 (GenBank number KR999961). The BS21 strain formed a cluster which was closely related to *B. subtilis* strains based on the neighbor-joining tree (Figure 2). Therefore, strain BS21 was identified as *B. subtilis* and named *B. subtilis* BS21. It was deposited in the China General Microbiological Culture Collection Center (strain No. 25977).

### 3.3. Genomic features of strain BS21

**Figure 3:**
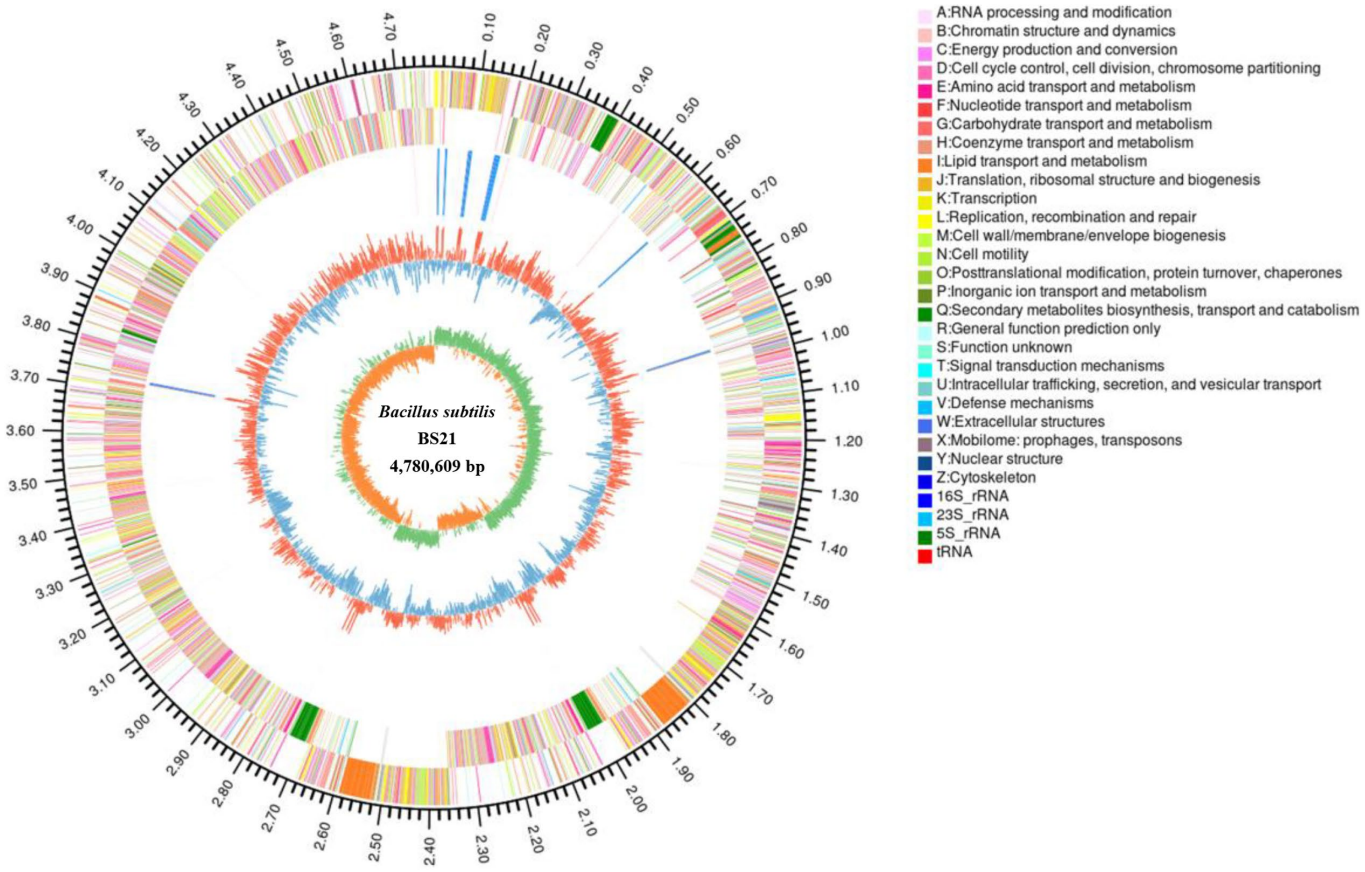
The circular genome map of strain BS21. From outside to inside, circle 1, the size of complete genome; circles 2 and 3, the predicted protein-coding genes on the positive and negative strands (different colors indicate different COG functions); circle 4, tRNA and rRNA; circle 5, GC content (red or blue represent the GC content higher or lower than average of GC content); the inner circle, GC-skew (green >0, yellow <0).

Genomic features of BS21 are represented in Figure 3. The complete genome of strain BS21 contained a circular chromosome with no plasmid. The genome size of BS21 was 4,780,609 bp and the GC content (guanine-cytosine percentage in the genome) was 43.85%. A total of 3,788 genes were identified that occupied 81.11% of the genome. Moreover, 4,670 protein CDSs, 30 rRNAs, 87 tRNAs, 1 prophage, 5 genomic islands and 4 CRISPR-Cas were predicted in the genome of strain BS21.

### 3.4. COG classification

**Figure 4:**
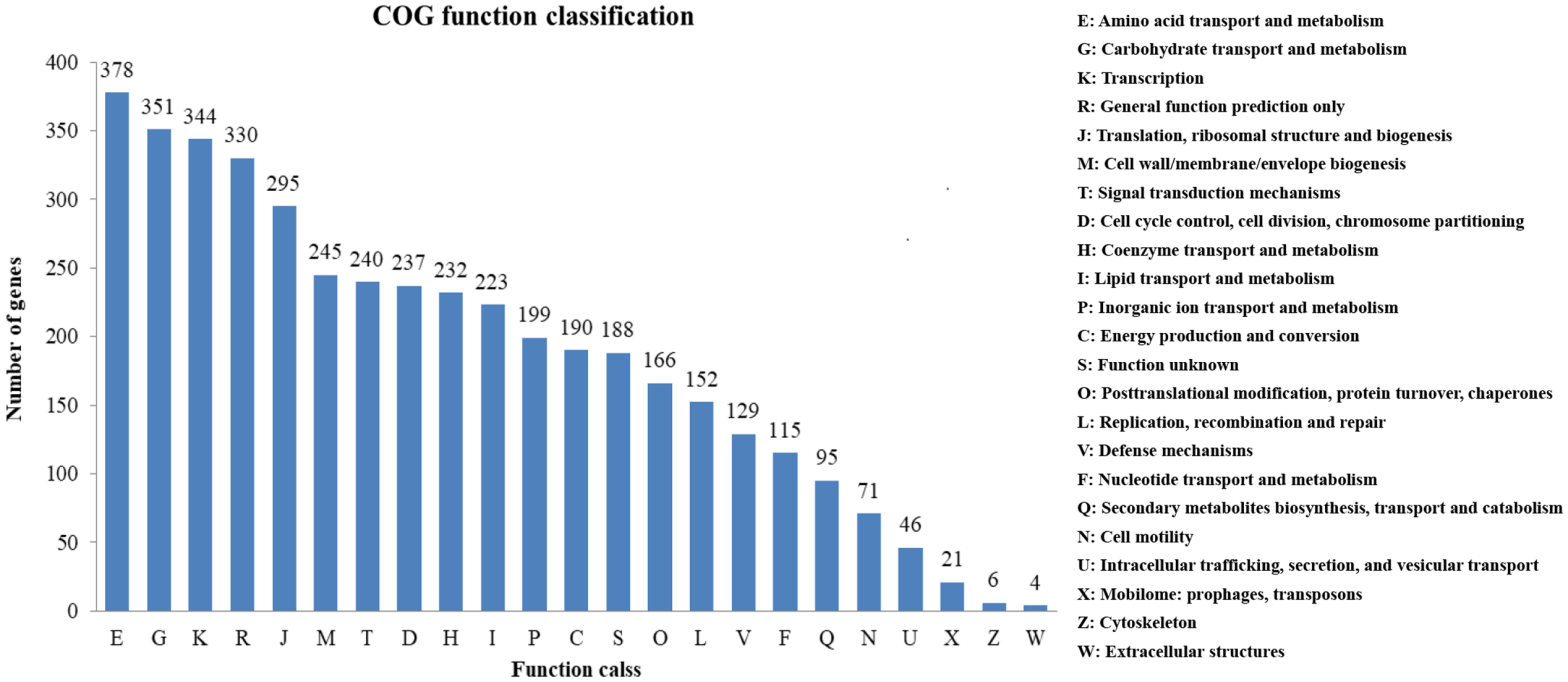
COG functional classification for strain BS21.

A high percentage of genes (81.11%) were annotated by COG. 23 COG functional categories contained a total of 3,788 genes (Figure 4). Many genes were classified into functional categories, including amino acid transport and metabolism (378 genes), carbohydrate transport and metabolism (351 genes), transcription (344 genes), general function prediction (330 genes) as well as translation, ribosomal structure and biogenesis (295 genes).

### 3.5. Genetic basis and UHPLC–MS/MS analysis for pathogen inhibition

**Figure 5:**
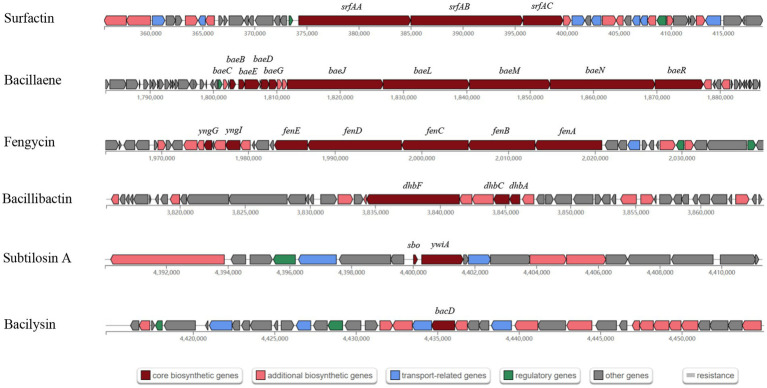
The antimicrobial secondary metabolite biosynthetic gene clusters in strain BS21. Red, pink, blue, green and gray indicate core biosynthetic genes, additional biosynthetic genes, transport related genes, regulatory genes and other genes, respectively. The core biosynthetic genes are marked in red.

**Figure 6:**
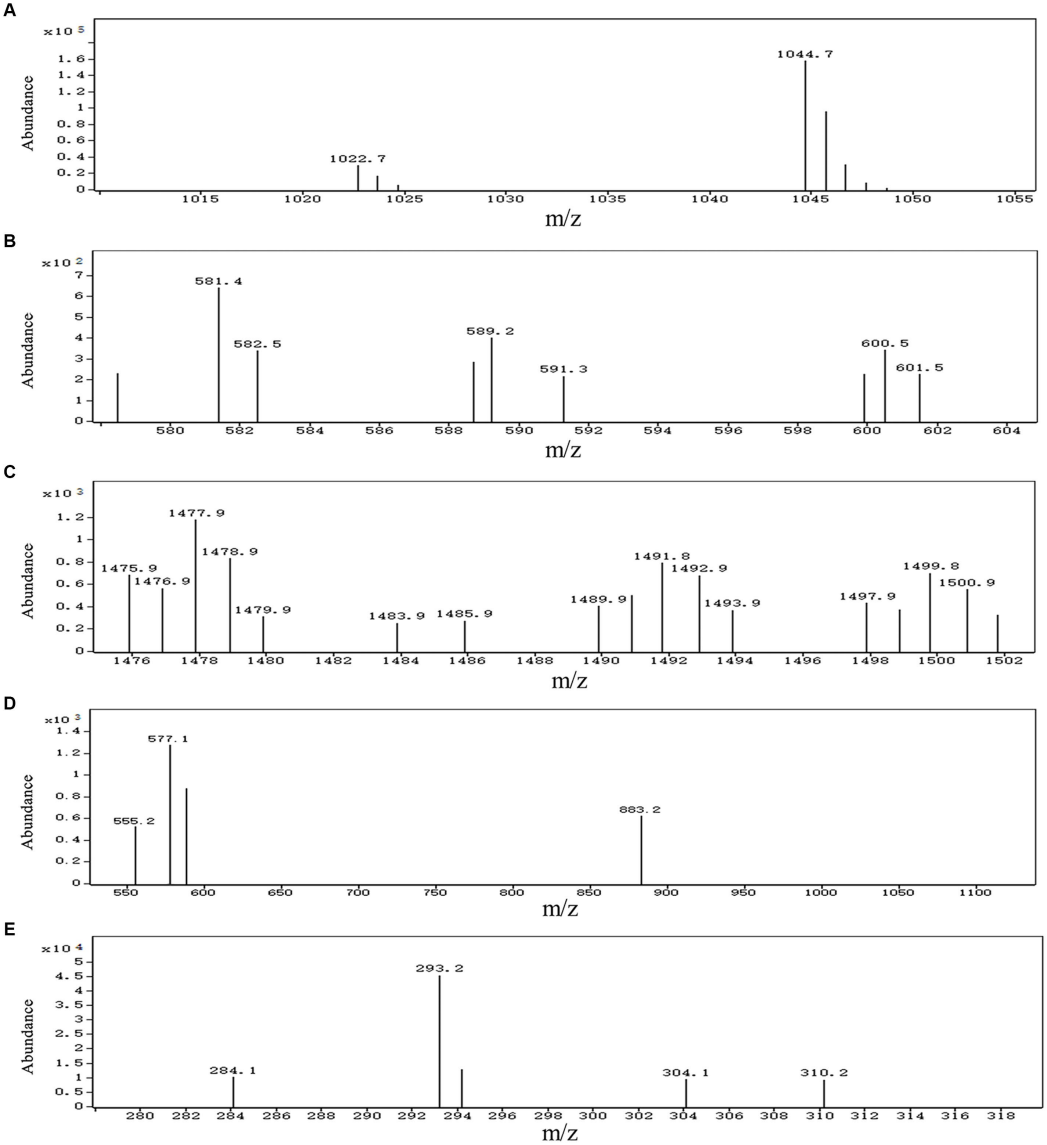
UHPLC–MS/MS analysis of antimicrobial secondary metabolites synthesized by strain BS21. **(A)** Ions of m/z values 1,022.7 correspond to C_14_ surfactin A [M + H]^+^, and ions of m/z values 1,044.7 correspond to C_14_ surfactin A [M + Na]^+^, respectively. **(B)** Ions of m/z 581.4 correspond to bacillaene A [M + H]^+^. **(C)** Ions of m/z values 1,477.9 correspond to C_15_ fengycin B [M + H]^+^, and ions of m/z values 1,499.8 correspond to C_15_ fengycin B [M + Na]^+^, respectively. **(D)** Ions of m/z 883.2 correspond to bacillibactin [M + H]^+^. **(E)** Ions of m/z value 293.2 correspond to bacilysin [M + Na]^+^.

A total of 13 gene clusters of secondary metabolites were predicted by antiSMASH in the genome of strain BS21, and seven were found to be associate with synthesis of antimicrobial secondary metabolites (Table 1). Among them, four gene clusters were predicted to synthesize the non-ribosomal peptides surfactin, zwittermicin A, bacillibactin and fengycin with 82, 22, 100 and 100% similarity, respectively. Two gene clusters had 100% similarity with one bacteriocin (subtilosin A) and one dipeptide (bacilysin). One gene cluster showed 100% similarity with a polyketide (bacillaene). These highly similar gene clusters (with more than 80% similarity) and their core genes are presented in Figure 5. Surfactin, bacillaene, fengycin, bacillibactin and bacilysin were further detected using UHPLC–MS/MS, verifying their successful production by BS21 (Figures 6A–E).

**Table 1:** Antimicrobial secondary metabolite clusters predicted in strain BS21.

No	Similar cluster	Cluster type	Similarity (%)	Size (kb)	Features	MIBiG accession
1	Surfactin	NRPS	82	63.27	Antibacterial, antifungal	BGC0000433
2	Zwittermicin A	NRPS	22	80.62	Antibacterial	BGC0001059
3	Unkown	Terpene	–	20.36	–	–
4	Bacillaene	TransAT-PKS	100	114.25	Antibacterial	BGC0001089
5	Fengycin	NRPS	100	82.17	Antifungal	BGC0001095
6	Unkown	Terpene	–	20.82	–	–
7	Unkown	T3PKS	–	40.65	–	–
8	Unkown	Terpene	–	21.90	–	–
9	Unkown	T3PKS	–	41.10	–	–
10	Bacillibactin	NRPS	100	49.74	Antibacterial, siderophore	BGC0000309
11	Unkown	Thiopeptide	–	30.11	–	–
12	Subtilosin A	Sactipeptide	100	21.61	Antibacterial	BGC0000602
13	Bacilysin	Other	100	41.42	Antibacterial, antifungal	BGC0001184

### 3.6. Optimization of fermentation conditions by single-factor experiment

**Figure 7:**
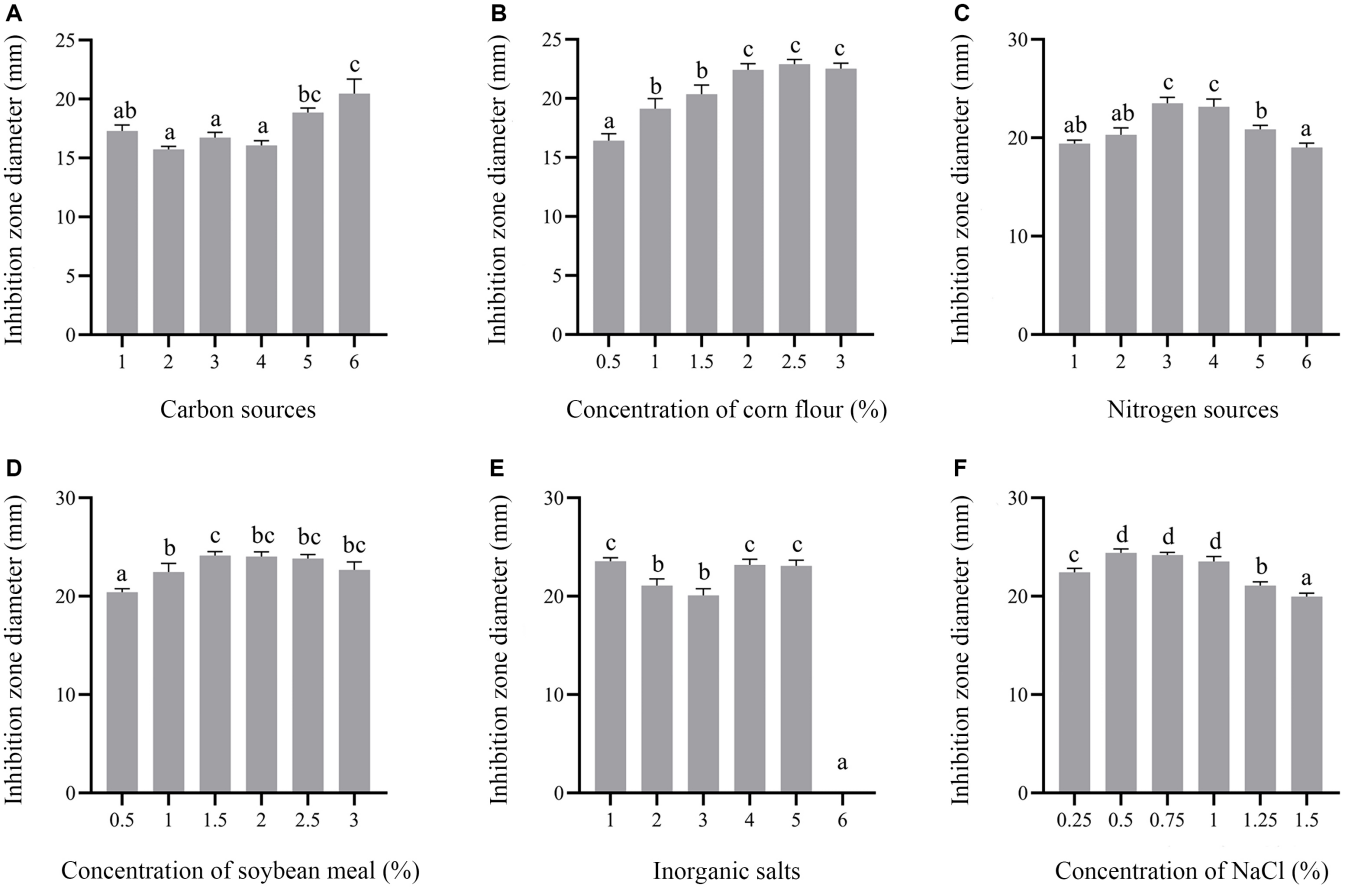
Effect of medium composition on antimicrobial secondary metabolite production. **(A)** Effect of different carbon sources (1%) (1: Glucose; 2: Fructose; 3: Sucrose; 4: Lactose; 5: Soluble starch; 6: Corn flour). **(B)** Effect of corn flour concentration. **(C)** Effect of different nitrogen sources (1%) (1: Yeast extract; 2: Tryptone; 3: Soybean meal; 4: Fermented soybean meal; 5: Rapeseed Meal; 6. Ammonium sulfate). **(D)** Effect of soybean meal concentration. **(E)** Effect of different mineral salts (1%) (1: NaCl; 2: CaCl_2_; 3: KH_2_PO_4_; 4. MgSO_4_; 5: ZnSO_4_; 6: MnSO_4_). **(F)** Effect of NaCl concentration on inhibition zone diameter.

**Figure 8:**
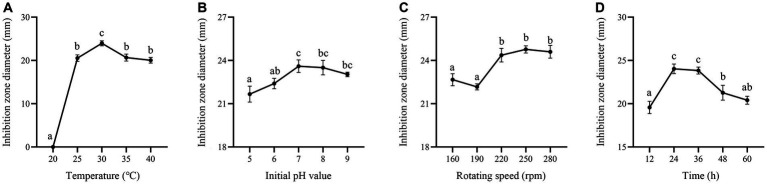
Effect of fermentation conditions on antimicrobial secondary metabolite production. **(A)** Effect of fermentation temperature. **(B)** Effect of initial pH value. **(C)** Effect of rotating speed. **(D)** Effect of fermentation time.

In this study, carbon sources, including glucose, fructose, sucrose, lactose, soluble starch and corn flour were supplemented in the culture medium. Supplementation of corn flour showed the maximum antimicrobial activity at an optimal concentration of 2% (Figures 7A,B). Yeast extract, peptone, soybean meal, fermented soybean meal, rapeseed meal and ammonium sulfate as sole nitrogen sources were added to culture strain BS21. The maximum inhibition zone diameter was detected when soybean meal was added as the carbon source, followed by fermented soybean meal with the optimal concentration of 1.5% (Figures 7C,D). Addition of MnSO_4_ led to the loss of antibacterial activity of strain BS21. The inhibition zone diameter in the NaCl group with the optimal concentration of 0.5% was greater than that in CaCl_2_, KH_2_PO_4_, MgSO_4_ and ZnSO_4_ groups (Figures 7E,F). Cell-free supernatant of strain BS21 showed no antimicrobial activity when cultured at 20°C, while the inhibition zone diameter was maximum at 30°C and decreased as temperature dropped (Figure 8A). Antimicrobial activity was high at pH 7.0 with a rotating speed of 220 rpm (Figures 8B,C). Inhibition zone diameter peaked at 24 h, indicating that antimicrobial secondary metabolites accumulated most at 24 h (Figure 8D).

### 3.7. Optimization of fermentation conditions by RSM

PBD is generally used to screen significant factors and evaluate their main effects. In this study, seven factors (corn flour, soya bean meal, NaCl, temperature, initial pH, rotational speed and fermentation time) were evaluated for their effect on antimicrobial activity of strain BS21. PBD and its experimental responses are shown in Table 2. The effect and significance of culture condition factors for PBD are shown in Table 3. The *p*-value in this model was below 0.05, suggesting that the regression equation carried statistical significance. The regression coefficient (R^2^) was 0.9731, demonstrating that 97.31% of the total variation could be explained by the developed model. The significance order of the seven tested factors on inhibition zone diameter was as follows: temperature > time > soybean meal > pH value > rotating speed > NaCl > corn flour, of which temperature, time and soybean meal were significant factors. Therefore, temperature, time and soybean meal were chosen for RSM to determine an optimum value and analyze their interactive effects.

**Table 2:** Design and results of Plackett-Burman experiment.

Run	A	B	C	D	E	F	G	Inhibition zone diameter (mm)
1	2	1.5	0.5	25	5	160	24	17.5
2	2	0.5	0.25	25	7	160	24	15.9
3	2	1.5	0.25	30	7	220	12	20.9
4	0.5	1.5	0.5	30	5	160	12	18.3
5	2	0.5	0.5	30	5	220	24	19.2
6	2	0.5	0.5	30	7	160	12	16.3
7	0.5	0.5	0.25	30	5	220	24	20.6
8	0.5	1.5	0.5	25	7	220	24	21.2
9	0.5	0.5	0.5	25	7	220	12	11.6
10	2	1.5	0.25	25	5	220	12	14.7
11	0.5	0.5	0.25	25	5	160	12	12.0
12	0.5	1.5	0.25	30	7	160	24	23.5

A = Corn flour (%), B = Soybean meal (%), C = NaCl (%), D = Temperature (°C), E = pH value, F = Rotating speed (rmp), G = Time (h). For each factor, the small values represent its low level (−1) and the large values represent its high level (+1).

**Table 3:** Analysis of variance for Plackett-Burman experiment.

Source	Sum of square	df	Mean square	*F*-value	*P*-value
Model	146.99	7	21	20.71	0.0054
A-Corn flour (%)	0.6075	1	0.6075	0.599	0.4822
B-Soybean meal (%)	35.02	1	35.02	34.53	0.0042
C-NaCl (%)	1.02	1	1.02	1.01	0.3725
D-Temperature (°C)	55.9	1	55.9	55.12	0.0018
E-pH value	4.2	1	4.2	4.14	0.1115
F-Rotating speed (rmp)	1.84	1	1.84	1.82	0.2492
G-Time (h)	48.4	1	48.4	47.72	0.0023
Residual	4.06	4	1.01		
Cor Total	151.05	11			

The *p*-values less than 0.05 are significant.

*R*^2^ = 0.9731, Adj *R*^2^ = 0.9261.

RSM had been proven to be a practical tool in the optimization of significant fermentation condition factors. In order to find out the optimum fermentation condition for production of antimicrobial secondary metabolites, BBD with three factors and three levels was adopted to optimize conditions further. BBD and its experimental responses are shown in Table 4. Analysis of variance for the response surface model is shown in Table 5. The *p*-value in this model was below 0.05, indicating that the regression equation was significant. The *R*^2^ was 0.9807, demonstrating that 98.07% of the total variation could be explained by the developed model. The response equation obtained was as follows:

**Table 4:** Design and results of Box–Behnken experiment.

Run	A-Soybean meal (%)	B-Temperature (°C)	C-Time (h)	Inhibition zone diameter (mm)
1	1.5	30	24	23.7
2	0.5	30	30	20.1
3	1.5	30	24	24.0
4	1.5	35	30	20.5
5	2.5	30	30	22.2
6	1.5	30	24	24.2
7	2.5	30	18	21.6
8	1.5	30	24	24.8
9	1.5	25	30	20.3
10	0.5	35	24	18.0
11	2.5	25	24	18.6
12	1.5	30	24	25.1
13	2.5	35	24	19.8
14	0.5	30	18	17.5
15	1.5	35	18	19.7
16	0.5	25	24	16.6
17	1.5	25	18	16.2

**Table 5:** Analysis of variance for the response surface model.

Source	Sum of square	df	Mean square	*F*-value	*p*-value
Model	130.12	9	14.46	39.46	<0.0001
A-Soybean meal (%)	12.5	1	12.5	34.12	0.0006
B-Temperature (°C)	4.96	1	4.96	13.54	0.0079
C-Time (h)	8.2	1	8.2	22.39	0.0021
AB	0.01	1	0.01	0.027	0.8734
AC	1	1	1	2.73	0.1425
BC	2.72	1	2.72	7.43	0.0295
A^2^	25.64	1	25.64	69.98	<0.0001
B^2^	55.86	1	55.86	152.49	<0.0001
C^2^	10.02	1	10.02	27.35	0.0012
Residual	2.56	7	0.37		
Lack of Fit	1.23	3	0.41	1.23	0.4072
Pure Error	1.33	4	0.33		
Cor Total	132.68	16			

The *p*-values less than 0.05 are significant.

*R*^2^ = 0.9807, Adj *R*^2^ = 0.9558.

$\begin{array}{l} Y=24.36+1.25^{*}A+0.79^{*}B+1.01^{*}C-0.05^{*}\mathrm{AB}\\ -0.5^{*}\mathrm{AC}-0.83^{*}BC-2.47^{*}A^{2}-3.64^{*}B^{2}-1.54^{*}C^{2} \end{array}$

**Figure 9:**
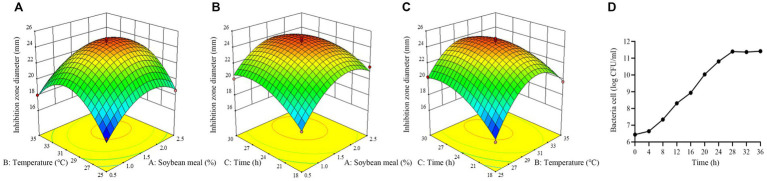
Response surface plot of the effect of three variables on antimicrobial activity of strain BS21. **(A)** Effect of soybean meal and temperature. **(B)** Effect of soybean meal and time. **(C)** Effect of temperature and time. **(D)** Time-dependent growth curve of strain BS21 under optimum condition.

The response surface plots showed that there is an interaction among three selected factors to affect antimicrobial activity of strain BS21 (Figures 9A–C). Moreover, the response surface was convex, meaning the existence of an optimal value for each variable. Based on our response surface analysis, the ideal values for the three tested factors were determined as: soybean meal at 1.7%, temperature at 30°C and time at 26 h. Under the optimal conditions, the inhibition zone diameter was predicted a theoretical maximum of 24.7 mm. The predicted values by RSM were confirmed through experimentation. At the optimum level, the inhibition zone diameter reached 25.1 mm, which was similar to the predicted maximum value, which showed a 43.4% increase compared to the original culture conditions (17.5 mm).

### 3.8. Growth curve of BS21 under optimum conditions

The growth curve of BS21 under optimum condition is presented as number of bacteria cells and showing a sigmoid growth pattern (Figure 9D). The rate of BS21 growth is slow at lag phase (0–4 h). The growth of BS21 is exponential from 4 to 28 h. After 28 h, BS21 transit to stationary phase, and the number of bacteria cell stabilized around 2.5 × 10^11^.

## 4. Discussion

Banning antibiotics in animal feed has resulted in reduced growth performance and higher morbidity and mortality. Probiotics are reported to improve animal health through stimulation of intestinal microbiota, host immunity and other factors (Ding et al., 2021). Their metabolites, also known as postbiotics, have strong activity against multiple pathogenic bacteria (Yadav et al., 2022). Therefore, probiotics and natural antimicrobial products from microorganisms seem to be a favorable alternative to antibiotics. Du et al. (2019) demonstrated that *B. subtilis* WS-1 inhibits the growth of *E. coli in vitro*, dietary supplementation with strain WS-1 significantly reduced diarrhea rates and death in piglets from *E. coli* infection and ameliorated small intestinal lesions. The yak feces-derived *B. velezensis* JT3-1 was strongly antagonistic against *E. coli*, *S. typhimurium*, *S. aureus* and *Mannheimia haemolytica*. The average weight, cure rate of diarrhea and levels of immunoglobulins (IgA, IgG, IgM) in Angus calves supplemented with *B. velezensis* JT3-1 were significantly improved (Li et al., 2019).

In this research, *Bacillus subtilis* BS21 isolated from pig feces exerted antibacterial activity on Gram-negative bacteria such as *E. coli*, *Salmonella*, *Shigella* and *Citrobacter*, which are major pathogens causing diarrhea in young animals (Li et al., 2021). In addition, strain BS21 also inhibited the growth of Gram-positive bacteria *S. aureus*, which can lead to mastitis in dairy cows (G Abril et al., 2020). These results indicated that strain BS21 has the potential to act as probiotic dietary supplements when added to animal feed and will show positive effects on animal health.

The strong antimicrobial activity of *Bacillus* species may be attributed to their property to produce multiple antimicrobial compounds, which favors their distribution in gut microbiota of animals to exert prebiotic functions (Caulier et al., 2019). A complete understanding in the genome of *B. subtilis* will contribute to the utilization of antimicrobial activity in strains of this species. The 43.85% GC content of BS21 was similar to previously reported *Bacillus subtilis* GM5 and 9407, which were 43.3 and 43.7%, respectively, (Hadieva et al., 2018; Gu et al., 2021). COG functional annotation of BS21 indicated that many genes are predicted to participate in amino acid transport and metabolism, carbohydrate transport, transcription as well as translation, ribosomal structure and biogenesis, which are important to the biological functionality of BS21. In addition, through antiSMASH tool and UHPLC–MS/MS analysis, secondary metabolites, including four antimicrobial peptides (surfactin, bacillibactin, fengycin, bacilysin) and one polyketide (bacillaene) were identified from culture supernatants, thus demonstrating that the corresponding biosynthetic gene clusters are functional in strain BS21.

Bacillibactin, a siderophore-type antimicrobial, show considerable antibacterial activity against *E. coli*, *S. aureus*, vancomycin-resistant *E. faecalis*, *P. aeruginosa*, and *K. pneumoniae* depending on its capability of complexing iron, which means that less iron was available to pathogens (Chen et al., 2019; Chakraborty et al., 2022). Fengycin, a lipopeptide, is known for its antifungal properties (Fu et al., 2022). In addition to directly restrict the growth of mycotoxigenic fungi, fengycin also inhibits mycotoxin production (Bertuzzi et al., 2022). Moreover, fengycin was reported by Piewngam et al. (2018) to eliminate *S. aureus* by blocking its quorum sensing. Surfactin is a common antimicrobial lipopeptide secreted by *Bacillus* species with antifungal, antiviral, anti-mycoplasma and antiprotozoal activities (Chen et al., 2022). The antibacterial mechanism of surfactin mainly relies on damage to bacterial cell membranes, inhibition to bacterial protein synthesis and bacterial enzyme activity (Theatre et al., 2021). It was reported that dietary surfactin supplementation improved growth performance and gut health of broilers challenged with *Clostridium perfringens* and tilapia (*Oreochromis niloticus*) fingerlings, suggesting surfactin as a potential substitute for antibiotics in poultry (Zhai et al., 2016; Cheng et al., 2018). In addition, surfactin also extends the shelf life of vegetables, fruits, meat, cereals and milk, functioning as a preservative with minimal degradability and toxicity (Huang et al., 2008; Kourmentza et al., 2021). Bacilysin is a simple antimicrobial peptide containing an L-alanine residue at the N-terminus and a non-proteinogenic amino acid L-anticapsin at the C-terminus (Islam et al., 2022). Although having a simple structure, it exhibits broad activity against various bacteria and fungi, attributing to its ability to induce cell lysis (Wang et al., 2018). Bacillaene, a polyene antibiotic, selectively inhibits prokaryotic protein synthesis (Olishevska et al., 2019). More valuably, these antimicrobial secondary metabolites may have synergistic effects against pathogens, therefore, have great potential to maintain microbiota homeostasis of intestine and regulate immune response of animals (Chen et al., 2019; Liu et al., 2022).

Production of antimicrobial secondary metabolites by microbial fermentation is strongly associated with culture conditions. Slight modifications in the composition of the medium or fermentation parameters can have a big impact on both the quality and quantity of secondary metabolites, also the overall metabolic characteristics of microorganisms (Sharma et al., 2020). Temperature can alter cell growth and product formation efficiencies (Vehapi et al., 2023). The temperature at which bacteria produce maximum antimicrobial secondary metabolites may not be the optimal temperature for their growth (Tan et al., 2022). It was reported that antibacterial compounds production of *Bacillus* spp. maximized at a temperature of 25–30°C (Puan et al., 2023). The antibacterial secondary metabolites produced by bacteria are reported to be synthesized during the exponential or early stationary phase (Boottanun et al., 2017; Regmi et al., 2017; Johny and Suresh, 2022). Therefore, optimizing fermentation conditions is a necessary strategy to develop biological additives. As a powerful mathematical tool for multiple regression analysis and statistical experimental designs, RSM has proven to be highly effective in optimizing formulation conditions (Mohammed and Luti, 2020). In this study, temperature, time and soybean meal were significant factors to effect antimicrobial activity of BS21 by screening of PBD. The optimal levels of them was predicted at 30°C, 26 h and soybean meal at 1.7% by BBD, respectively. The maximum antimicrobial activity of BS21 was predicted at the end of the exponential phase of growth (at 26 h) by BBD experiment. The significant interactions among temperature, time and soybean meal were obtained from the response surface plots.

In our investigation, the diameter of inhibition zone reached 25.1 mm after optimization using single-factor experiments and RSM with BBD, representing a 43.4% increase compared to the original formulation conditions. Similarly, RSM had let to improved production of antibacterial compounds in different bacterial strains. In a recent study by Sa et al. (2022), the formulation conditions of *Paenibacillus polymyxa* DS-R5 strain were optimized by using RSM experimental design. This optimized conditions (medium volume 51.0 mL; initial pH 6.7; fermentation temperature 33.1°C) led to a remarkable 77.6% increase in the titer of antifungal substances, with the production reaching 8036 mg/L compared to the initial formulation conditions of 4357 mg/L. Ju et al. (2018) demonstrated that the inhibition zone diameter of cell-free culture supernatant of the *Streptomyces rimosus* AG-P1441 strain was enhanced from an initial 15 to a 29 mm under the optimized culture conditions (3% glucose, 3.5% corn starch, 2.5% soybean meal, 1.2 mM MgCl_2_ and 5.9 mM glutamate) by using RSM. As a result, RSM showed a high accuracy of the developed model and model validation under the present study, it could be a reliable way to optimize the formulation conditions for antimicrobial secondary metabolites of the BS21 strain.

In conclusion, strain BS21 and its antimicrobial secondary metabolites have the potential to be developed as antibiotic alternatives to control pathogenic bacteria and improve growth performance of animals. Optimization studies using the single-factor design and RSM successfully increased the production of antimicrobial secondary metabolites of strain BS21. This study not only lays an experimental basis for subsequent development and application of strain BS21 as an antibiotic alternative in animal production, but also provides a reference for improving the production of antimicrobial secondary metabolites in other bacterial strains.

## Data availability statement

The complete genome sequence of Bacillus subtilis BS21 has been deposited in GenBank (Accession number PRJNA962810).

## Author contributions

DW, ND, and YC conceived and designed the research. DW and LF performed the experiments. ND and DL contributed analytical tools and reagents. DW wrote the manuscript. All authors have read and approved the manuscript.

## Footnotes

^1^ https://github.com/hyattpd/Prodigal

^2^ http://topaz.gatech.edu/GeneMark

^3^ http://trna.ucsc.edu/software/

^4^ https://github.com/tseemann/barrnap/

^5^ http://www.pathogenomics.sfu.ca/islandviewer/

^6^ http://phast.wishartlab.com/

^7^ http://crispr.i2bc.paris-saclay.fr/

^8^ https://github.com/bbuchfink/diamond

^9^ https://ftp.ncbi.nlm.nih.gov/blast/executables/blast+/LATEST/

^10^ http://www.hmmer.org/

^11^ https://dl.secondarymetabolites.org/releases/4.0.2/
